# The guanine nucleotide exchange factor Vav3 intervenes in the migration pathway of oligodendrocyte precursor cells on tenascin-C

**DOI:** 10.3389/fcell.2022.1042403

**Published:** 2022-11-30

**Authors:** Ina Schäfer, Juliane Bauch, David Wegrzyn, Lars Roll, Simon van Leeuwen, Annika Jarocki, Andreas Faissner

**Affiliations:** Department of Cell Morphology and Molecular Neurobiology, Ruhr University Bochum, Bochum, Germany

**Keywords:** extracellular matrix, glia, laminin, migration, oligodendrocyte precursor cell, Rho GTPase, tenascin, Vav3

## Abstract

Oligodendrocyte precursor cells (OPCs) are the exclusive source of myelination in the central nervous system (CNS). Prior to myelination, OPCs migrate to target areas and mature into myelinating oligodendrocytes. This process is underpinned by drastic changes of the cytoskeleton and partially driven by pathways involving small GTPases of the Rho subfamily. In general, the myelination process requires migration, proliferation and differentiation of OPCs. Presently, these processes are only partially understood. In this study, we analyzed the impact of the guanine nucleotide exchange factor (GEF) Vav3 on the migration behavior of OPCs. Vav3 is known to regulate RhoA, Rac1 and RhoG activity and is therefore a promising candidate with regard to a regulatory role concerning the rearrangement of the cytoskeleton. Our study focused on the *Vav3* knockout mouse and revealed an enhanced migration capacity of *Vav3* ^−/−^ OPCs on the extracellular matrix (ECM) glycoprotein tenascin-C (TnC). The migration behavior of individual OPCs on further ECM molecules such as laminin-1 (Ln1), laminin-2 (Ln2) and tenascin-R (TnR) was not affected by the elimination of Vav3. The migration process was further investigated with regard to intracellular signal transmission by pharmacological blockade of downstream pathways of specific Rho GTPases. Our data suggest that activation of RhoA GTPase signaling compromises migration, as inhibition of RhoA-signaling promoted migration behavior. This study provides novel insights into the control of OPC migration, which could be useful for further understanding of the complex differentiation and myelination process.

## Introduction

Myelin formation in the central nervous system (CNS) is achieved by oligodendrocytes, the second largest population of macroglia in the brain (Nave and Werner, 2014; Valerio-Gomes et al., 2018). Prior to myelination, the oligodendrocyte precursor cells (OPCs) migrate from their site of origin through the CNS and differentiate into mature oligodendrocytes (Barateiro and Fernandes, 2014). This process is guided by multiple factors such as gradients of the bone morphogenetic protein (BMP) or sonic hedgehog (SHH), even during demyelination and remyelination (Ferent et al., 2013; Petersen et al., 2017). In addition, local factors are of huge interest in scientific research. In this context, the extracellular matrix (ECM) and its various components comprise promising candidates to guide cell migration as substrates. Here, we focused on laminins-1 and -2 (Ln1, Ln2) and tenascins-C and -R (TnC, TnR). Previous studies on TnC have revealed an inhibitory influence of the molecule on OPCs migration, myelin-basic protein (MBP) expression and, finally, also on myelination and remyelination (Kiernan et al., 1996; Wiemann et al., 2020; Bauch et al., 2022). Comparable functions have been demonstrated for TnR (Huang et al., 2009; Bauch et al., 2022). In contrast, laminins promote OPC migration *in vitro* (Frost et al., 1996; Milner et al., 1996; Suzuki et al., 2019). Recognition of these ECM molecules is achieved by specific transmembrane receptors such as the integrins, which have the ability to interact intracellularly with small GTPases. Furthermore, changes of the actin-cytoskeleton due to migration and later differentiation are partially driven by small GTPases, in particular the RhoA-family that therefore plays an important role in oligodendrocyte development. It has been shown that the differential activation of RhoA, Rac1 and Cdc42 alters the differentiation behavior of oligodendrocytes (Liang et al., 2004; Czopka et al., 2009; Ulc et al., 2019). Furthermore, there is evidence that Rho activation in fibroblasts promotes stress fiber creation and activation of Rac1 leads to filopodia and lamellipodia formation (Nobes and Hall, 1995). These GTPases are switches in signal pathways and can be either “on” or “off” by cycling between a GTP-bound or GDP-bound state. Key regulators of this cycle are on the one hand GTPase-activating proteins (GAPs), which enhance the intrinsic GTPase activity and thereby turn the switch “off” and on the other hand guanine nucleotide exchange factors (GEFs), which catalyze the exchange of GDP to GTP to switch a pathway “on” (Govek et al., 2005; Bos et al., 2007; Ulc et al., 2017).

Vav3 is the newest family member of the Vav-gene family of Rho GEFs, which was identified in 1999 (Movilla and Bustelo, 1999). Vav1 is restricted to the hematopoetic system, whereas Vav2 and Vav3 are more ubiquitously expressed (Hornstein et al., 2004) and show a somewhat broader distribution, including neural tissues (Ulc et al., 2019; Wegrzyn et al., 2020). Vav family members share a very similar domain structure. All isoforms contain an auto-inhibitory acid motif, which represses the GEF activity until the protein is phosphorylated (Turner and Billadeau, 2002). Furthermore they contain a Dbl homology domain, which is a hallmark for all Rho GEFs (Zheng, 2001), that promotes guanine nucleotide exchange and SH2 and SH3 domains to bind to phosphorylated tyrosine residues (Jaffe and Hall, 2005). Activation of Vav *via* phosphorylation can be achieved by multiple mechanisms including receptor tyrosine kinases such as the EGF-receptor (EGFR) (Bustelo et al., 1992; Margolis et al., 1992), the IGF1R (Uddin et al., 1995), the PDGFR (Bustelo et al., 1992) and TrkA (Melamed et al., 1999; Bustelo, 2014). It has been shown that the EGFR specifically activates Vav3 (Moores et al., 2000). As a unique feature among Rho-GEFs the Vav proteins are auto-inhibited under resting conditions and activated by the phosphorylation of tyrosine residues in the acidic region (Yu et al., 2010). Upstream, membrane-bound tyrosine-kinase receptors (RTKs) as well as cytoplasmic TKs such as src can activate the GEF-function, which places Vav proteins at an intersection point between extracellular signals and intracellular differentiation processes (Bustelo, 2001; Turner and Billadeau, 2002; Bustelo, 2014; Ojala et al., 2020). Of particular interest for developmental neuroscience, EGF and PDGF can turn on Vav proteins *via* their membrane-bound RTKs (Moores et al., 2000; Chiariello et al., 2001; Marcoux and Vuori, 2003). The cytokines EGF and PDGF are potent mitogens of neural stem cells and OPCs, respectively (Kriegstein and Alvarez-Buylla, 2009; Bergles and Richardson, 2016; Ulc et al., 2017).

Vav3-GEF activity is directed to the small GTPases RhoA, RhoG and to lesser extent Rac1 and Cdc42 (Movilla and Bustelo, 1999; Sachdev et al., 2002; Aoki et al., 2005; Toumaniantz et al., 2009). RhoA and Rac1 are important for the formation of myelin membranes in oligodendrocytes and RhoA, Rac1 and Cdc42 are expressed in the oligodendrocyte lineage (Czopka et al., 2009; Rajasekharan et al., 2010). It has also been shown that Vav3 promotes vascular smooth muscle cell migration through activation of Rac1/Pak signaling (Toumaniantz et al., 2009). Here, we investigate the role of Vav3 during oligodendrocyte precursor migration using a recently described *Vav3* knockout (*Vav3* ^−/−^) mouse (Luft et al., 2015). Using time-lapse video microscopy, we identified an accelerated migration velocity of *Vav3* ^−/−^ OPCs on the extracellular matrix molecule tenascin-C (TnC). Furthermore, a compromising effect of RhoA signaling and a promoting effect of Rac1 were observed that affect different migration parameters.

## Materials and methods

### Animals

The *Vav3* knockout mouse line was originally produced using the targeting vector combined with embryonic stem (ES) cells of the mouse strain 129/Sv, as described previously (Luft et al., 2015). The *Vav3* knockout mouse line carries a mixed genetic background of C57Bl/6 and 129/Sv mouse strains (assessed by GVG Genetic Monitoring GmbH, Leipzig, Germany). *Vav3* knockout mice were backcrossed to the C57BL/6J strain for 5 generations. Mice were bred and housed according to institutional guidelines. Genomic DNA was genotyped by PCR using a *Vav3* forward primer 5′- GCA CTC GCT GCT GCA GCG GC -3′ and a *Vav3* Exon 1 reverse primer 5′- GAG AAA CTG GGA CAT CTG GGG CCT CAG -3′ to generate a fragment of approximately 400 bp wild-type DNA; alternatively, the *Vav3* forward primer and the NEO primer 5′- CAG GTA GCC GGA TCA AGC GTA TGC -3′ were applied to generate a 600 bp fragment and thereby detect *Vav3* knockout DNA (primers by Sigma-Aldrich, St. Louis, United States). All experiments were carried out using *Vav3* ^+/+^ and *Vav3* ^−/−^ littermates of a heterozygous breeding of *Vav* ^+/-^ animals.

### Culturing of oligospheres

OPCs were generated out of neurospheres integrating the intermediate step of oligosphere formation, as previously described (Pedraza et al., 2008). The cortices of embryonic day (E) 13.5–15.5 mice were dissected and enzymatically digested using 30 U/ml papain (Cat. No: LS003126, Worthington, Columbia, United States) and 0.24 mg/ml L-cysteine (Cat. No: C252, Sigma-Aldrich) for 25 min at 37°C. The digestion was stopped with stopping solution [trypsin inhibitor (Cat. No: T6522, Sigma-Aldrich), BSA 5% (w/v) (Cat. No: A4919, Sigma-Aldrich), DNase 0.5% (w/v) (Cat. No: LS0020007, Worthington) in Leibovitz´s L-15-medium (Cat. No: L5520; Sigma-Aldrich)] and the cortices were mechanically triturated. The single cell suspension was then cultivated in neurosphere media [DMEM/F12 (Cat. No: 2,417,144, Thermo Fisher Scientific, Waltham, United States), 2% (v/v) B27 (Cat. No: 17,504,044, Thermo Fisher Scientific), 1% (v/v) penicillin/streptomycin (P/S) (Cat. No: P4333, Sigma-Aldrich)] supplemented with epidermal growth factor (EGF, 20 ng/ml, Cat. No: 100–15, Peprotech, Rocky Hill, United States) and incubated at 37°C and 6% (v/v) CO_2_. Spheres were passaged every 2–3 days. After 5 days, the neurosphere medium was exchanged against culture medium supplemented with platelet-derived growth factor (PDGF, 20 ng/ml, Cat. No: 100-13A, Peprotech) and fibroblast growth factor (FGF-2, 20 ng/ml, Cat. No: 100-18B, Peprotech), instead of EGF. Another 5 days later, the oligospheres were plated as whole spheres for the time-lapse migration assay.

### Time-lapse migration assay

The time-lapse migration assay was carried out with the Axiovert 200 M (Zeiss, Oberkochen, Germany). Oligospheres were plated in 24-well plates with different coatings. As control condition a poly-D-lysine (PDL) coating was used (10 μg/ml, Cat. No: P0899, Sigma-Aldrich). To analyze the influence of coating with ECM proteins, coating with laminin-1 (Ln1; 10 μg/ml, Cat. No: 354,259, Sigma-Aldrich), laminin-2 (Ln2; 10 μg/ml, Cat. No: L0663, Sigma-Aldrich), tenascin-C (TnC; 40 μg/ml, purified from neonatal mouse brains; Faissner and Kruse, 1990), or tenascin-R (TnR; 40 μg/ml, purified from adult mouse brains; Czopka et al., 2009) was performed following a precoating with poly-L-ornithine (PLO) (15 μg/ml, Cat. No: P3655; Sigma-Aldrich). For 24 h the Axiovision 4.8 software (Zeiss) acquired an image every 8 min at one defined position in each well (as shown in exemplary videos Supplementary Videos 1–7). During this procedure the temperature was maintained constant at 37°C and the CO_2_ at 7.5%. Following the acquisition, the images were analyzed by using the ImageJ (NIH, Bethesda, United States) software (Schneider et al., 2012) and the “Manual Tracking” plugin. At least 5 far migrated cells per sphere were tracked over the 24 h timeframe (as shown in Supplementary Videos 1, 2). Data was expressed as migration speed in µm per h (µm/h).

### Counting cells and measuring halo distances

Using ImageJ/FIJI (NIH, Bethesda, United States) software (Schindelin et al., 2012) and the “Cell Counter” plugin, the number of migrated cells during 24 h of migration assay was investigated and depicted in total numbers. At least 4 and at most 487 cells were observed outside the oligosphere. Furthermore, the plugin “Concentric Circles” of ImageJ/FIJI software was used to analyze the halos of migrated cells. This distance is defined as the distance from the edge of the sphere (inner circle) to the outer circle that touched the farthest migrated cell. Measured halo distances were expressed in µm. Here, distances between 88 and 686 µm were quantified.

### Blockage of GTPases with the inhibitors Y27632 and EHT 1864

To investigate intracellular downstream interaction partners of Vav3, blockage of signaling pathways mediated *via* RhoA and Rac1 was performed. Therefore, time-lapse video microscopy was performed with plated oligospheres, which were treated with specific inhibitors, namely Y27632 (inhibitor of the RhoA-associated kinase ROCK; Cat. No: 129,830–38–2, Abcam, Berlin, Germany) and EHT 1864 (inhibitor of Rac1; Cat. No: 754,240–09–0, Merck, Darmstadt, Germany). The OPC culture medium [DMEM (Cat. No: 2,425,936, Thermo Fisher Scientific), 1% (v/v) N2 (Cat. No: 17,502,048, Thermo Fisher Scientific), 1% (v/v) P/S (Cat. No: P4333, Sigma-Aldrich), 1% (w/v) BSA (Cat. No: A4919, Sigma-Aldrich), 20 ng/ml PDGF-AA (Cat. No: 100-13A, Peprotech)] was supplemented with 10 µM Y27632 (Czopka et al., 2009) or 5 µM EHT 1864 (Shutes et al., 2007). Oligospheres were plated on PDL-, Ln1- or TnC-coated 24-well plates as previously described and were already incubated in presence or absence (control condition) of the inhibitors one hour prior to and during 24 h of time-lapse video microscopy.

### Documentation and data analysis

Time-lapse images were taken with a Axiovert 200 M equipped with the AxioCam HRm using Axiovision 4.8 software (Zeiss, Oberkochen, Germany). Statistical analysis is depicted as the mean ± SEM. In case of time-lapse migration assay, all single data points are also represented in the graphs. “N” defines the number of biological replicates and “n” stands for technical replicate numbers. Migration Assay: *N* = 5, *n* = 25; cell numbers: *N* = 5, *n* = 5; halo distance: *N* = 5, *n* = 5; blockage migration assay: *N* = 5, *n* = 25; except: PDL controls (the same set of OPCs used for the control condition served as reference for all experiments with laminins and tenascins, including inhibition approaches): *N* = 10, *n* = 50 (migration velocity in migration assay and inhibition assay). For a better overview and to allow a direct comparison with the control condition in different contexts, we show the control condition also in the following figures (indicated by identical representative images and data sets).

The normal distribution of values was examined with the Shapiro-Wilk test. In case of normal distribution, statistical significance was assessed using ANOVA and Bonferroni Post-test, subsequently. Statistical significances of not normally distributed values were investigated using non-parametric ANOVA, Kruskal–Wallis test and Dunn’s post test, subsequently. In some cases, we saw differences between wild-type and Vav3-deficient OPC conditions, where no significant alterations were observed with the ANOVA test, but with Student’s *t*-test (TnC, migration speed, Figures 4, 6).

For the cell number and halo distance analysis only Mann Whitney *U*-test or Wilcoxon matched pairs test (referring to the suggestion of the statistics program) were applied. The *p*-values are given as *p ≤ 0.05, ** 0.01 ≥ *p* ≥ 0.001, ***p ≤ 0.001. All statistical tests were performed with GraphPad Prism (Graphpad Software, La Jolla, United States).

## Results

### Migration speed of oligodendrocyte precursor cells on laminins

**FIGURE 1:**
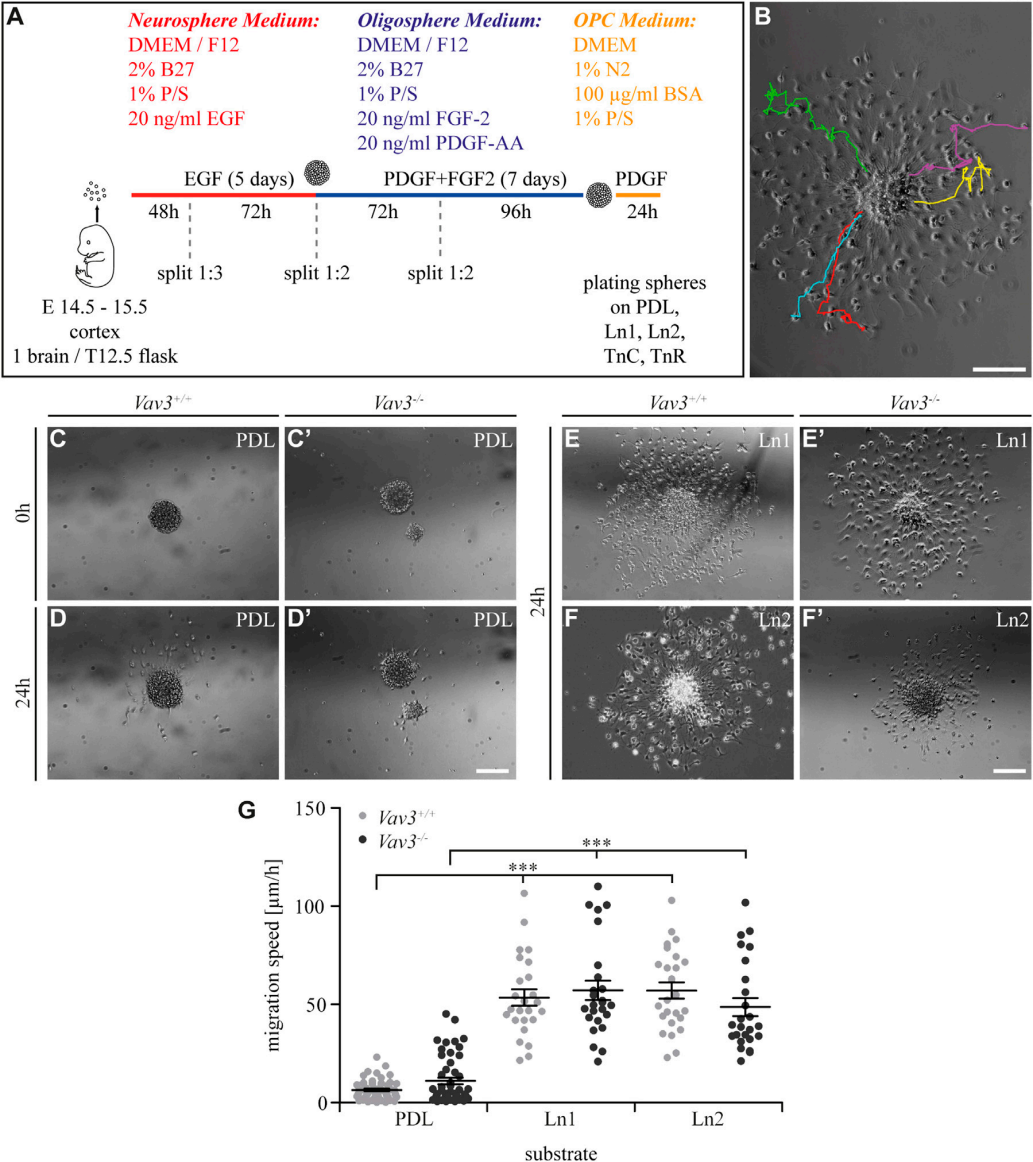
Generation of oligospheres and migration speed of OPCs on PDL and laminins. **(A)** Protocol for the generation of OPCs and the migration assay. **(B)** Representative image of a plated oligosphere with tracked cells and their individual traces. **(C + C′)** Plated Vav3-expressing (*Vav3* ^+/+^) and Vav3-deficient (*Vav3* ^−/−^) oligospheres on PDL substrate (control) at timepoint 0 h. **(D + D′)** Plated oligospheres of either genotype on PDL after 24 h of migration time. **(E + E′)** Oligospheres of either genotype on laminin-1 (Ln1) substrate after 24 h of migration. **(F + F′)** Both genotypes of oligospheres plated on laminin-2 (Ln2) after 24 h of migration. **(G)** The quantification of migration speed [in µm/h] revealed no genotype-dependent effect of OPCs on different substrates but indicated a significantly increased migration velocity of OPCs on laminins compared to the control PDL. Data are expressed as mean ± SEM. Single values are depicted as data points. ANOVA was performed and depending on normal distribution of values, Bonferroni post test or Kruskal–Wallis and Dunn’s post test followed (***p ≤ 0.001). PDL: *N* = 10, *n* = 50; laminins: *N* = 5, *n* = 25. Scale: 200 µm.

OPCs are the source of mature myelinating oligodendrocytes and have to be highly migratory, as their main function is to populate the brain, proliferate in the target compartment and differentiate into mature oligodendrocytes. As their migration potential and capacity is interesting for potential therapeutic approaches, we established a novel way of investigating this cellular function. Therefore, we performed time-lapse video microscopy with previously generated cortical oligospheres on different extracellular matrix molecules (Figure 1A; Supplementary Videos 1–7). Rho GTPases are known to be involved in mediating migration wherefore we used our Vav3-deficient mouse model to identify a connection between Rho GTPase signaling, the GEF Vav3 and the extracellular substrate.

In our first experiment, we focused on laminin coatings, laminin-1 (Ln1) and laminin-2 (Ln2) respectively. Oligospheres were plated on control coating PDL and the laminins before the cellular behavior in response to the substrate was analyzed by video microscopy. We focused on the five farthest migrated cells and measured the velocity of the moving OPCs (Figure 1B). Compared to the control condition PDL (Figures 1C, C′, D, D′, G) the OPCs on both laminins were significantly faster (Figures 1E, E′, F, F′, G) (WT: PDL 6.5 ± 1.6 μm/h SEM, Ln1 53.5 ± 9.3 μm/h SEM, Ln2 57.0 ± 9.3 μm/h SEM; KO: PDL 11.0 ± 3.8 μm/h SEM, Ln1 57.2 ± 11.1 μm/h SEM, Ln2 48.7 ± 10.2 μm/h SEM; ***p ≤ 0.001) (compare Supplementary Videos 3–7). This demonstrated a faster OPC migration on laminins compared to the control. However, Vav3 had no impact on the migration velocity in this set-up.

**FIGURE 2:**
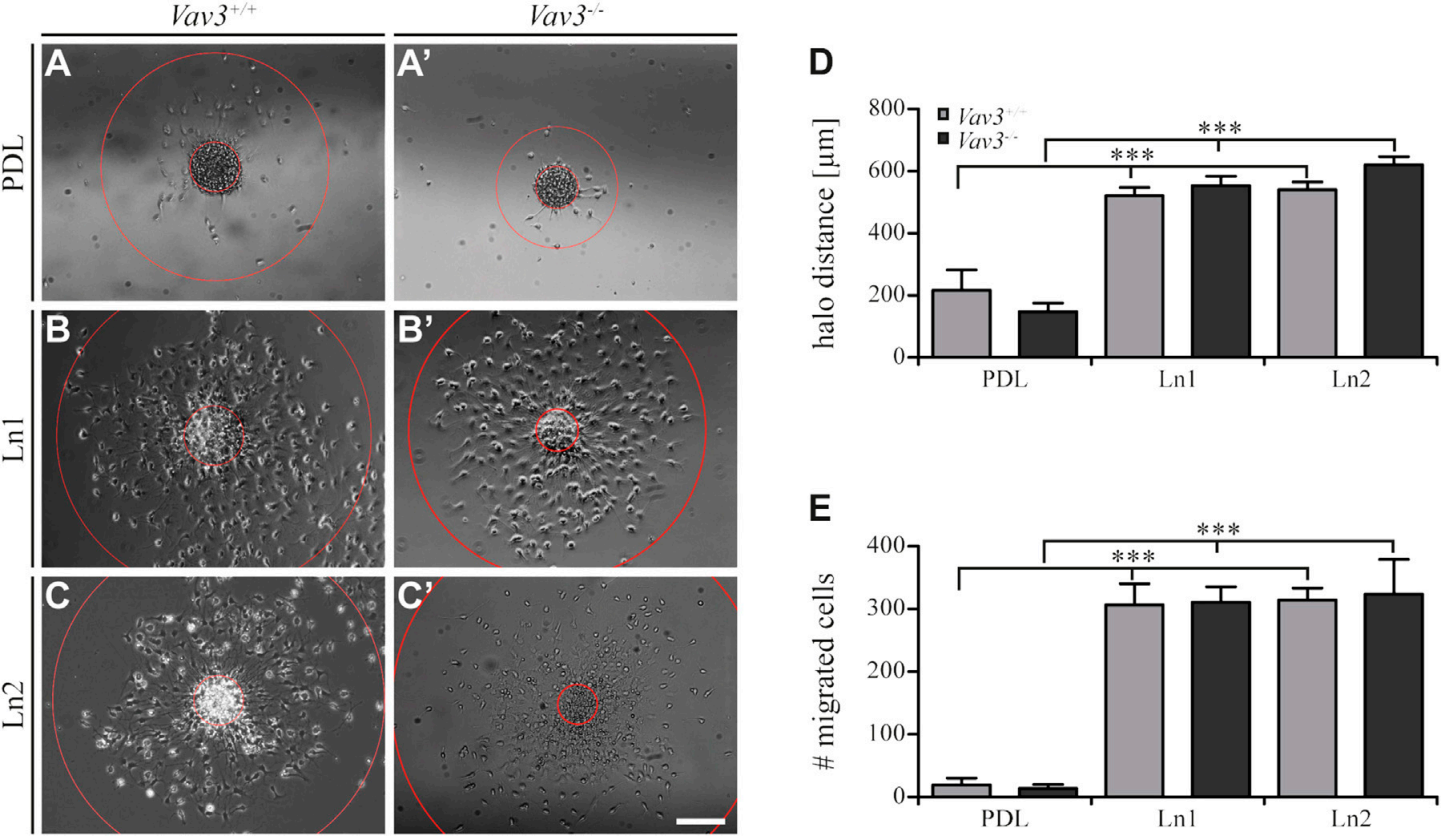
Migration of OPCs from oligospheres on PDL and laminins. **(A + A′)** Vav3-expressing (*Vav3* ^+/+^) and Vav3-deficient (*Vav3* ^−/−^) oligospheres on PDL after 24 h of migration. **(B + B′)** Successful migration of OPCs from oligospheres of either genotype on laminin-1 (Ln1) after 24 h. **(C + C′)** Migration success of OPCs from *Vav3* ^+/+^ and *Vav3* ^−/−^ oligospheres on laminin-2 (Ln2) after 24 h. **(D)** The quantification of halo distance [in µm] revealed that OPCs on laminins covered significantly longer distances compared to the OPCs on the control substrate. **(E)** The quantification of total cell numbers (#) migrating on the different substrates again demonstrated a significantly higher number of moving OPCs on laminins compared to the control. Data is expressed as mean ± SEM. Student’s *t*-test was performed (***p ≤ 0.001). *N* = 5, *n* = 5. Scale: 200 µm.

By analyzing the migration speed, only a part of the data was calculated. In another approach, we concentrated on the radius or halo, in which the OPCs moved. Based on the farthest migrated cell, which defined the outer halo, and the edge of the sphere, which defined the inner halo (Figure 2A–C′), we observed significantly increased distances [in µm] on both laminin conditions (WT: PDL 217.2 ± 65.2 µm SEM, Ln1 520.9 ± 26.9 µm SEM, Ln2 540.4 ± 24.9 µm SEM; KO: PDL 147.6 ± 28.3 µm SEM, Ln1 553.7 ± 30.2 µm SEM, Ln2 620.6 ± 26.7 µm SEM; ***p ≤ 0.001). This indicated that OPCs on laminins not only migrated faster (Figure 1), but also covered a greater distance compared to the control condition PDL (Figure 2D).

Additionally, we also investigated the number of migrated cells under the different conditions and found similar results considering the radius parameter. Again, oligospheres on laminins showed significantly increased numbers of migrated OPCs (Figure 2E) that left the plated oligosphere compared to the control PDL (WT: PDL 19.4 ± 10.7 SEM, Ln1 306.6 ± 33.7 SEM, Ln2 314.2 ± 18.9 SEM; KO: PDL 13.8 ± 6.4 SEM, Ln1 310.4 ± 25.0 SEM, Ln2 323.8 ± 55.2 SEM; ***p ≤ 0.001).

In conclusion, we could see that laminins enhanced the migration speed, migration radius and number of migrated cells in comparison to the PDL control. However, these effects appeared to be independent from the GEF Vav3, as we did not observe differences between the two genotype conditions.

### Inhibition of signaling along Rho GTPases RhoA and Rac1 in Vav3-deficient OPCs in comparison to the control on laminin

**FIGURE 3:**
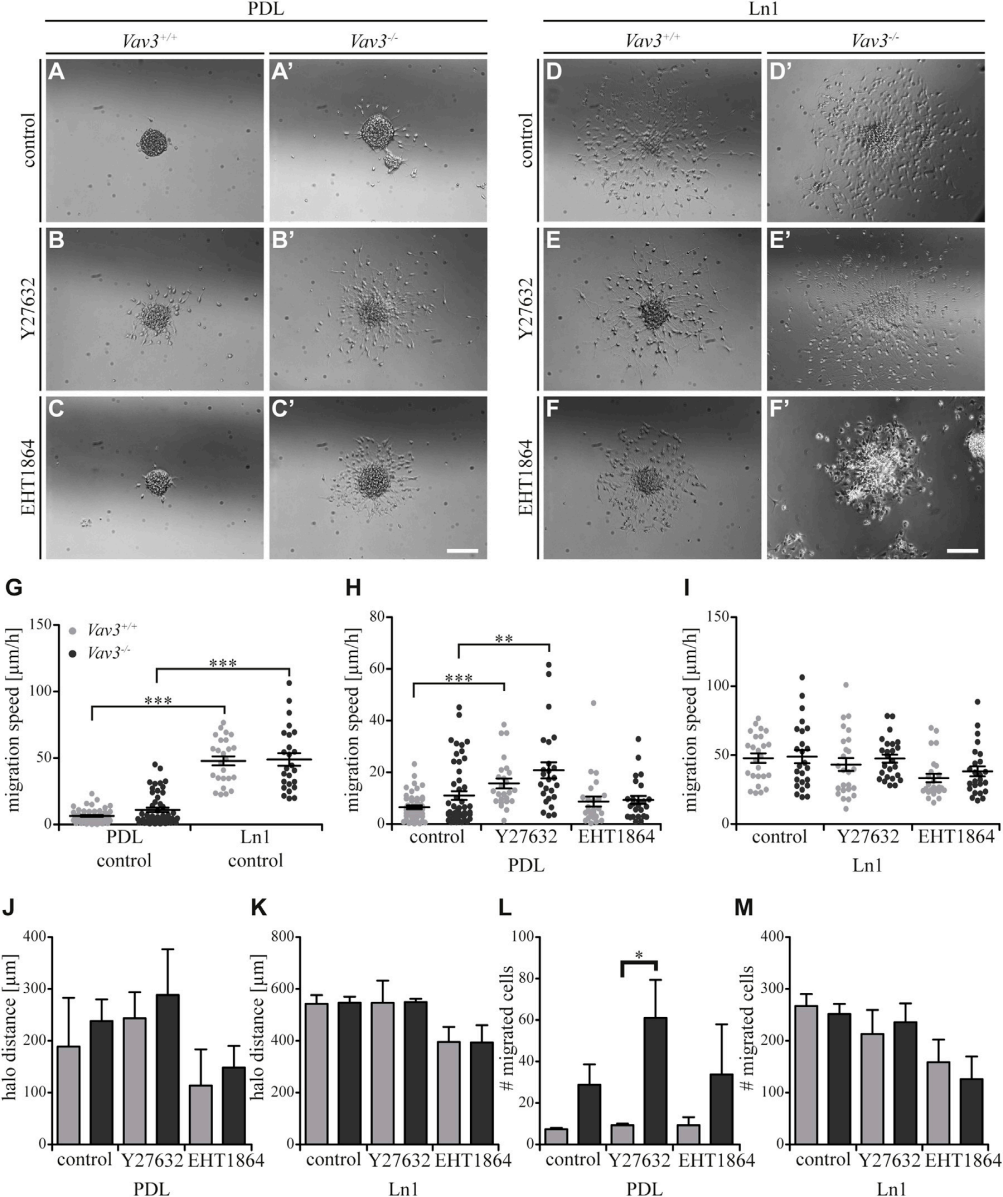
Effect of inhibited Rho GTPase signaling on OPC migration on PDL and laminin-1. **(A + A′)** Control condition of oligospheres of either genotype on PDL without treatment (after 24 h). **(B + B′)** Blockage of ROCK by treatment with Y27632 inhibitor in oligospheres of both genotypes on PDL after 24 h. **(C + C′)** Blockage of Rac1 by treatment with EHT 1864 inhibitor in oligospheres of both genotypes on PDL after 24 h. **(D + D′)** Control condition of oligospheres of either genotype on Ln1 without treatment (after 24 h). **(E + E′)** Blockage of ROCK by treatment with Y27632 inhibitor in oligospheres of both genotypes on Ln1 after 24 h. **(F + F′)** Blockage of Rac1 by treatment with EHT 1864 inhibitor in oligospheres of both genotypes on Ln1 after 24 h. **(G)** Quantified migration speed of PDL and Ln1 control conditions without treatment. Significantly impaired migration speed was observed on PDL compared to Ln1 in both genotypes. **(H)** The quantification of the migration speed of OPCs from oligospheres of either genotype on PDL indicated a RhoA-related migration behavior, independent of Vav3. **(I)** The quantification of migration speed of OPCs from oligospheres of either genotype on Ln1 indicated a very mild involvement of Rac1, as migration speed was not heavily affected by Rac1 inhibition. **(J)** The halo distance slightly supported a restrained involvement of RhoA in migration behavior of OPCs of either genotype on PDL. Inhibition of Rac1 caused a decreased halo distance that was statistically not significant. **(K)** The halo distance of OPCs of either genotype supported the involvement of Rac1 in migration behavior. Although the changes were not statistically significant. **(L)** The number of migrated cells (#) on PDL demonstrated low numbers in *Vav3* ^+/+^ OPC cultures. In comparison the numbers of migrated *Vav3* ^−/−^ cells were increased in every condition, with a significant effect after ROCK inhibition. **(M)** The number of migrated cells (#) on Ln1 revealed a slight, non-significant decrease after EHT 1864 treatment, again supporting the observation of an influence of Rac1 in migration behavior of OPCs of either genotype on Ln1. Most effects seen were independent from the *Vav3* genotype. Data are expressed as mean ± SEM. ANOVA was performed and depending on normal distribution of values, Bonferroni post test or Kruskal–Wallis and Dunn’s post test followed. Additionally, Student’s *t*-test was performed for halos and cell numbers. *p ≤ 0.05, ** 0.01 ≥ *p* ≥ 0.001, ***p ≤ 0.001. Migration assay: PDL control *N* = 10, *n* = 50, PDL and Ln1 *N* = 5, *n* = 25; halos and cell numbers: *N* = 5, *n* = 5. Scale: 200 µm.

Depending on the substrate and the receptors, such as integrins, that recognize the molecules, different Rho GTPases can be intracellularly activated and induce specific signaling cascades. Here, we analyzed the influence of two inhibitors of GTPases, which may be activated by laminins depending on the GEF Vav3. Y27632 blocks ROCK as a downstream effector of RhoA GTPase, whereas EHT 1864 inhibits Rac1 GTPase (Figure 3).

As mentioned above, we could show that OPCs migrate significantly faster on Ln1 compared to PDL control condition without inhibitor treatment (Figure 3G, compare Figure 1, ***p ≤ 0.001). Focusing on the control substrate PDL we identified that inhibition of the RhoA GTPase pathway in Vav3-deficient and Vav3-expressing cells resulted in an increased migration speed of cells of either genotype, whereas inhibition of Rac1 GTPase had no significant impact on the migration speed (WT: control 6.5 ± 1.6 μm/h SEM, Y27632 15.7 ± 4.3 μm/h SEM, EHT 1864 8.8 ± 4.4 μm/h SEM; KO: control 11.0 ± 3.8 μm/h SEM, Y27632 20.8 ± 7.0 μm/h SEM, EHT 1864 9.3 ± 3.6 μm/h SEM; ** 0.01 ≥ *p* ≥ 0.001, ***p ≤ 0.001) (Figure 3H). This was also seen in both genotypes. Using the same experimental set-up, now with Ln1 as substrate, we saw a slightly decreased migration speed of OPCs through the inhibition with EHT 1864 (WT: control 47.9 ± 7.7 μm/h SEM, Y27632 43.2 ± 10.4 μm/h SEM, EHT 1864 33.4 ± 6.8 μm/h SEM; KO: control 48.9 ± 10.6 μm/h SEM, Y27632 47.5 ± 6.7 μm/h SEM, EHT 1864 38.3 ± 8.1 μm/h SEM) (Figure 3I). However, this effect was not significant and was observed independently from presence of the GEF Vav3.

Additionally, we analyzed the halo distance under inhibition conditions and saw a slight increase after ROCK inhibition and in contrast a slight decrease after Rac1 blockage on PDL (WT: control 188.7 ± 94.2 µm SEM, Y27632 243.3 ± 50.4 µm SEM, EHT 1864 113.8 ± 69.8 µm SEM; KO: control 238.0 ± 41.8 µm SEM, Y27632 288.6 ± 88.0 µm SEM, EHT 1864 148.6 ± 41.6 µm SEM) (Figure 3J). The number of migrated cells also indicated an increase after Y27632 treatment and additionally a significant difference between Vav3-deficient OPCs and control cells (WT: control 7.3 ± 0.7 SEM, Y27632 9.3 ± 0.7 SEM, EHT 1864 9.3 ± 3.8 SEM; KO: control 28.8 ± 9.8 SEM, Y27632 61.0 ± 18.3 SEM, EHT 1864 33.8 ± 24.2 SEM; *p ≤ 0.05) (Figure 3L).

On Ln1, longer distances were covered compared to PDL and blockage with the Rac1 inhibitor revealed a (not significantly) reduced radius in cell cultures of both genotypes compared to the control (WT: control 543.3 ± 33.0 µm SEM, Y27632 546.4 ± 85.8 µm SEM, EHT 1864 395.3 ± 58.3 µm SEM; KO: control 546.8 ± 23.9 µm SEM, Y27632 549.6 ± 12.6 µm SEM, EHT 1864 393.6 ± 67.0 µm SEM) (Figure 3K). Also, the number of moving OPCs appeared smaller after Rac1 inhibition with EHT 1864 compared to the control condition. As for the halo radius, this observation was not statistically significant (WT: control 267 ± 23.0 SEM, Y27632 213.0 ± 46.5 SEM, EHT 1864 158.7 ± 43.6 SEM; KO: control 251.8 ± 19.4 SEM, Y27632 236.0 ± 36.2 SEM, EHT 1864 126.0 ± 43.4 SEM) (Figure 3M). Here we demonstrate that blockage of ROCK and therewith inhibition of the RhoA GTPase pathway influenced the migration behavior in cases of migration speed, halo radius and number of migratory cells to a certain degree, however these effects were not significant. Most of the observations were independent from the *Vav3* knockout. However, it is worth noting that the number of migrated cells on PDL after Y27632 treatment was significantly higher in Vav3-deficient oligospheres, indicating an inhibitory effect of RhoA-activation in Vav3-dependent migration behavior on PDL. Moreover, we observed tendential effects of Rac1 inhibition on OPC migration, especially on Ln1, but these events were independent from Vav3 deficiency and statistically not significant.

### Migration speed of OPCs on tenascins

**FIGURE 4:**
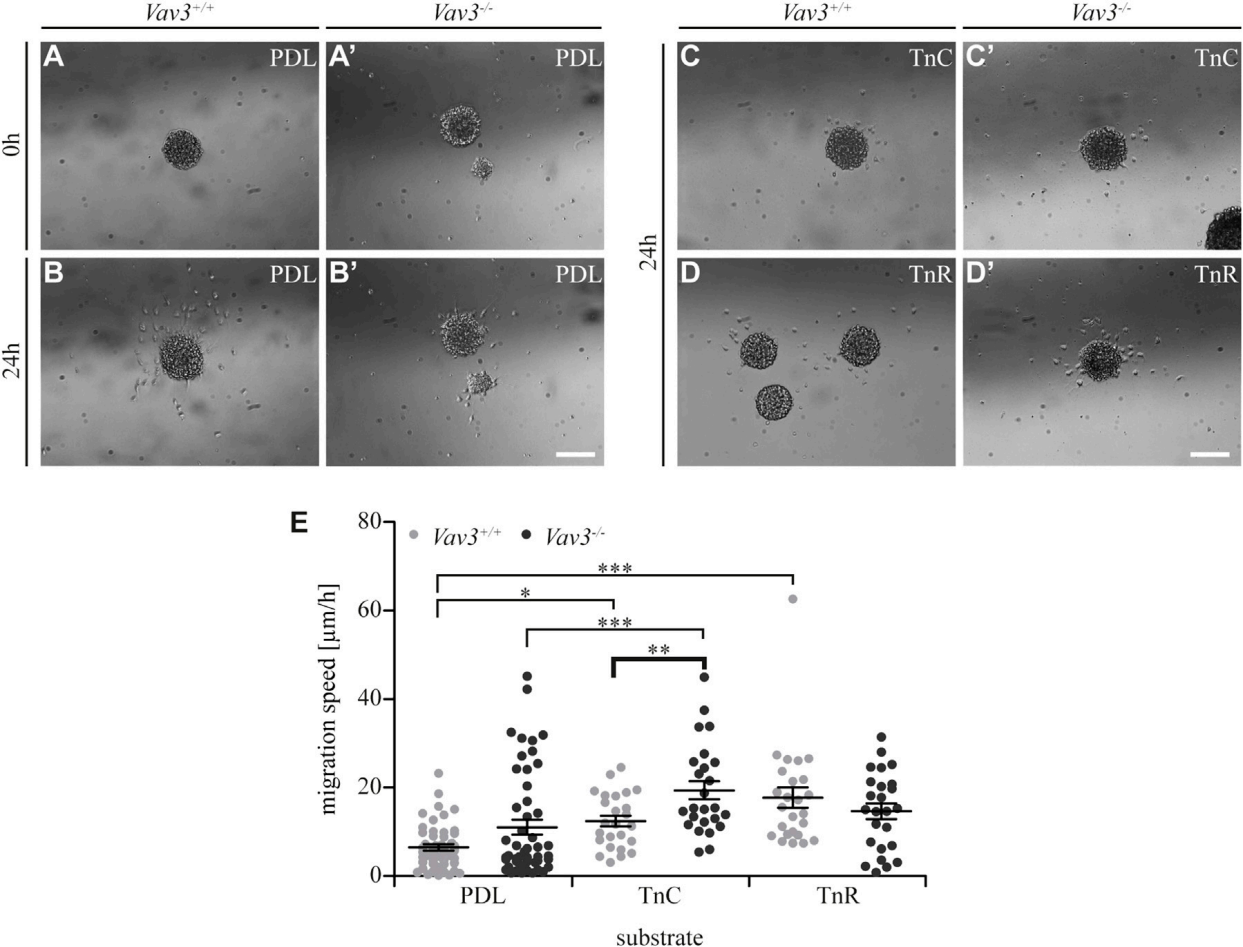
Migration speed of OPCs from oligospheres on PDL and tenascins. **(A + A′)** Plated Vav3-expressing (*Vav3* ^+/+^) and Vav3-deficient (*Vav3* ^−/−^) oligospheres on PDL substrate (control) at timepoint 0 h. **(B + B′)** Plated oligospheres of either genotype on PDL after 24 h of migration time. **(C + C′)** Oligospheres of either genotype on tenascin-C (TnC) substrate after 24 h of migration. **(D + D′)** Oligospheres of both genotypes plated on tenascin-R (TnR) after 24 h of migration. **(E)** The quantification of migration speed [in µm/h] revealed not only significantly increased migration velocities on tenascins compared to PDL, but also a significant, Vav3-dependent effect on TnC: *Vav3* ^−/−^ OPCs migrated significantly faster than *Vav3* ^+/+^ OPCs. Data are expressed as mean ± SEM. Single values are depicted as data points. ANOVA was performed and depending on normal distribution of values, Bonferroni post test or Kruskal–Wallis and Dunn’s post test followed (***p ≤ 0.001; ** 0.01 ≥ *p* ≥ 0.001; *p ≤ 0.05). PDL: *N* = 10, *n* = 50; tenascins: *N* = 5, *n* = 25. Scale: 200 µm.

Next, we further focused on the extracellular matrix molecules tenascin-C (TnC) and tenascin-R (TnR). Using video microscopy, we investigated the migration velocity of Vav3-deficient and wild-type OPCs on tenascin substrates (Figure 4; Supplementary Videos 1, 2, 6, 7). Obviously, OPCs barely migrated on tenascins, and clearly not as extensively as on laminins (compare Figure 1), consistent with earlier reports. Yet, the migration speed appeared to be slightly increased compared to the control, except for Vav3-deficient cells on TnR (WT: PDL 6.5 ± 1.6 μm/h SEM, TnC 12.4 ± 2.8 μm/h SEM, TnR 17.7 ± 5.2 μm/h SEM; KO: PDL 11.0 ± 3.8 μm/h SEM, TnC 19.4 ± 4.6 μm/h SEM, TnR 14.7 ± 4.0 μm/h SEM; *p ≤ 0.05, ***p ≤ 0.001) (Figure 4E). This might reflect the known anti-adhesive properties of tenascins for OPCs (Pesheva et al., 1989; Bartsch et al., 1994). Additionally, we found a striking effect of Vav3 ablation with regard to the migration behavior of Vav3-deficient OPCs on TnC. Here, we recorded a significantly increased speed of Vav3-deficient OPCs compared to the wild-type control (WT: 12.4 ± 2.8 μm/h SEM, KO: 19.4 ± 4.6 μm/h SEM; ** 0.01 ≥ *p* ≥ 0.001). Thereby, the migration speed appeared increased by 56.5%. This data indicated a strongly compromised OPC migration on TnC when Vav3 was expressed and active in the cell and demonstrated a major relation between TnC and Vav3-related GTPase signaling.

**FIGURE 5:**
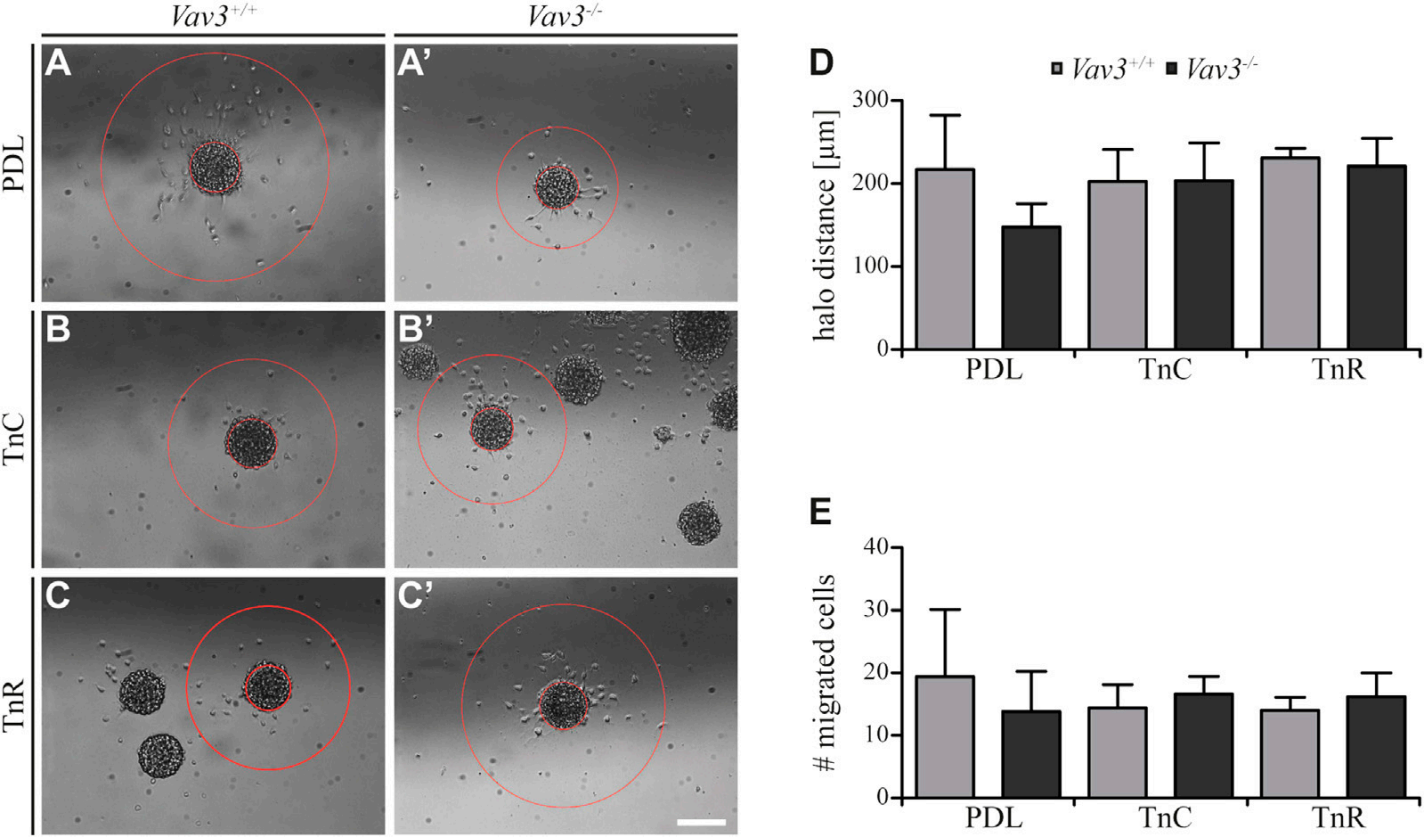
Migration of OPCs from oligospheres on PDL and tenascins. **(A + A′)** Vav3-expressing (*Vav3* ^+/+^) and Vav3-deficient (*Vav3* ^−/−^) oligospheres on PDL after 24 h of migration. **(B + B′)** Migration success of OPCs from oligospheres of either genotype on tenascin-C (TnC) after 24 h. **(C + C′)** Migration success of OPCs from *Vav3* ^+/+^ and *Vav3* ^−/−^ oligospheres on tenascin-R (TnR) after 24 h. **(D)** The quantification of halo distance [in µm] revealed that OPCs on tenascins covered similar distances as OPCs on PDL. **(E)** The quantification of total cell numbers (#) migrating on the different substrates again demonstrated similar numbers of moving OPCs on tenascins compared to the control. Data are expressed as mean ± SEM. Student’s *t*-test was performed. *N* = 5, *n* = 5. Scale: 200 µm.

Completing this analysis, we next focused on the halo distances and number of migrating cells on tenascin substrates (Figure 5). Compared to the PDL control, the radius of migrating OPCs on TnC and TnR was similar (WT: PDL 217.2 ± 65 µm SEM, TnC 202.4 ± 38.4 µm SEM, TnR 230.8 ± 11.8 µm SEM; KO: PDL 147.6 ± 28.3 µm SEM, TnC 203.6 ± 45.5 µm SEM, TnR 221.2 ± 33.2 µm SEM) (Figure 5D). Furthermore, the numbers of OPCs that left the plated oligosphere were comparable in the TnC, TnR and control conditions (WT: PDL 19.4 ± 10.7 SEM, TnC 14.4 ± 3.7 SEM, TnR 14.0 ± 2.1 SEM; KO: PDL 13.8 ± 6.4 SEM, TnC 16.6 ± 2.8 SEM, TnR 16.2 ± 3.8 SEM) (Figure 5E). However, these observed effects were Vav3-independent, as WT and *Vav3* knockout did not differ.

Although we noted a strong relation of Tnc and GEF Vav3 signaling indicated by an increased migration velocity in Vav3-deficient oligospheres, the distance that was covered and the number of cells that migrated were not affected. The similar radius contrasted with a faster migration rate, which could be explained by cells that moved in more “zigzag” directions rather than on straight radial paths.

### Inhibition of signaling along Rho GTPases RhoA and Rac1 in Vav3-deficient OPCs in comparison to the control on tenascin

**FIGURE 6:**
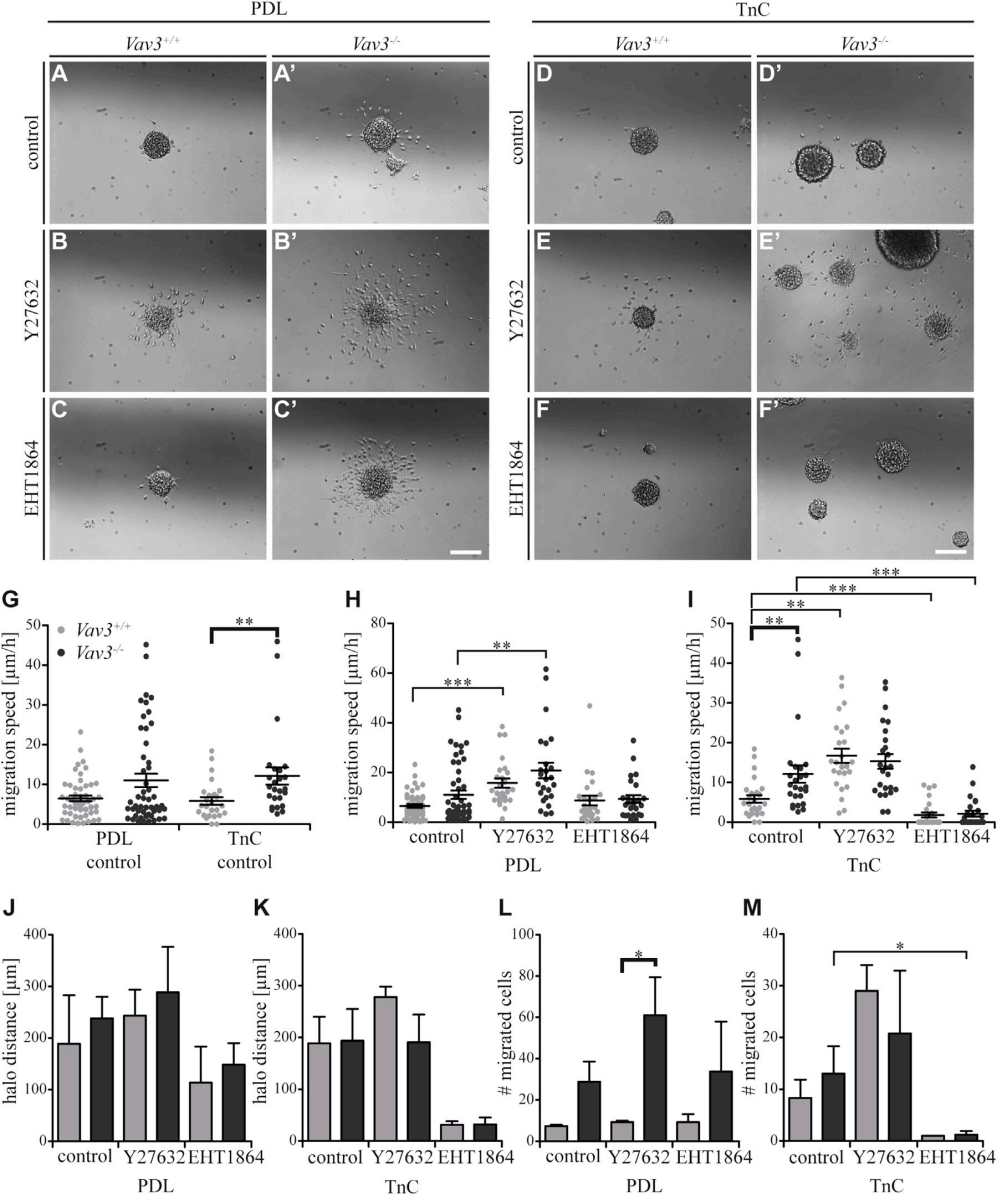
Effect of inhibited Rho GTPase signaling on OPC migration on PDL and tenascins. **(A + A′)** Control condition of oligospheres of either genotype on PDL without treatment (after 24 h). **(B + B′)** Blockage of ROCK by treating oligospheres of both genotypes on PDL with the inhibitor Y27632 after 24 h. **(C + C′)** Blockage of Rac1 by a treatment of oligospheres of both genotypes on PDL with the inhibitor EHT 1864 after 24 h. **(D + D′)** Control condition of oligospheres of either genotype on TnC without treatment (after 24 h). **(E + E′)** Blockage of ROCK by a treatment of oligospheres on TnC with the inhibitor Y27632 after 24 h. **(F + F′)** Blockage of Rac1 by treating oligospheres of both genotypes on TnC with the inhibitor EHT 1864 after 24 h. **(G)** Quantified migration speed of PDL and TnC control conditions without treatment. A significantly lower migration speed was observed for *Vav3* ^+/+^ OPCs compared to *Vav3* ^−/−^ on TnC, whereas velocities on PDL compared to TnC were similar in general. **(H)** The quantification of the migration speed of OPCs indicated a RhoA related migration behavior on PDL, with significantly higher velocities seen after ROCK inhibition in both genotypes. **(I)** The quantification of the migration speed of OPCs from oligospheres of either genotype on TnC indicated a strong Rac1-dependent and also RhoA-related migration speed. **(J)** The halo distance slightly supported a restrained involvement of RhoA in migration behavior of OPCs of either genotype on PDL. Inhibition of Rac1 caused a decreased halo distance that was statistically not significant. **(K)** The halo distance of OPCs of either genotype supported the involvement of Rac1 in migration behavior on TnC. Also here the effect was not statistically significant. **(L)** The number of migrated cells (#) on PDL demonstrated low numbers of migrated *Vav3* ^+/+^ OPCs. In comparison, the numbers of migrated *Vav3* ^−/−^ cells were increased in every condition, with a significant difference between both genotypes after ROCK inhibition. **(M)** The number of migrated cells (#) on TnC revealed a strong decrease after EHT 1864 treatment, again supporting the observation of an influence of Rac1 on migration behavior of OPCs of either genotype on TnC. Data are expressed as mean ± SEM. ANOVA was performed and depending on normal distribution of values, Bonferroni post test or Kruskal–Wallis and Dunn’s post test followed. Additionally, Student’s *t*-test was performed for halos and cell numbers and additionally for TnC migration speed. *p ≤ 0.05, ** 0.01 ≥ *p* ≥ 0.001, ***p ≤ 0.001. Migration assay: PDL control *N* = 10, *n* = 50, PDL and Ln1 *N* = 5, *n* = 25; halos and cell numbers: *N* = 5, *n* = 5. Scale: 200 µm.

Completing the data set for TnC, we analyzed the effect of blockage of Rho GTPase pathways (Figures 6A–F’). As previously mentioned, we could observe a significantly higher migration speed of Vav3-deficient OPCs on TnC (Figure 6G). Compared to the OPCs on the PDL control substrate, similar results were observed for TnC, which indicated that both substrates affected the migration speed of wild-type or Vav3-deficient OPCs in a comparable way (Figure 6G). The results for PDL (Figure 6H) have already been described in the context of laminin-1 (Figure 3H).

Blockage of Rho GTPase signaling in OPCs on TnC yielded significant alterations. The inhibition of the RhoA GTPase-dependent Rho kinase ROCK translated into a significantly higher velocity of wild-type OPCs and to a slightly increased velocity of Vav3-deficient OPCs on PDL (WT: control 5.9 ± 2.2 μm/h SEM, Y27632 16.7 ± 4.0 μm/h SEM; KO: control 12.1 ± 4.9 μm/h SEM, Y27632 15.3 ± 4.1 μm/h SEM; ** 0.01 ≥ *p* ≥ 0.001; Figure 6I). This data supported the conclusion that the RhoA-pathway seemed to restrain the migratory behavior. Interestingly, both the ablation of Vav3 and the inhibition of RhoA-dependent pathways similarly enhanced the migration velocity on TnC (Figures 6G,I). However, the application of the ROCK inhibitor did not enhance further the migratory activity of *Vav3* ^*−/−*^ OPCs beyond the rate observed without blockade. This suggests that Vav3 exerts the major control of RhoA and downstream ROCK activation in this situation (Figure 6I). We conclude therefore that the activation of RhoA *via* Vav3 is responsible for the slowed migration on the TnC glycoprotein.

Different from this situation, a significantly reduced migration speed was observed after EHT 1864 treatment applied to interfere with Rac1 GTPases, pointing to a positive influence of Rac1 on the migration behavior of OPCs of either genotype on the TnC substrate (WT: control 5.9 ± 2.2 μm/h SEM, EHT 1864 1.8 ± 1.4 μm/h SEM; KO: control 12.1 ± 4.9 μm/h SEM, EHT 1864 2.2 ± 1.6 μm/h SEM; ***p ≤ 0.001) (Figure 6I). Referring to this observation, complementary data regarding the halo distance and the number of migrated cells supported our findings (halos: WT: control 188.7 ± 51.3 µm SEM, Y27632 278.0 ± 20.5 µm SEM, EHT 1864 31.1 ± 7.2 µm SEM; KO: control 193.9 ± 61.3 µm SEM, Y27632 190.8 ± 53.7 µm SEM, EHT 1864 32.0 ± 13.5 µm SEM; cell numbers: WT: control 8.3 ± 3.5 SEM, Y27632 29.0 ± 5.0 SEM, EHT 1864 1.0 ± 0.0 SEM; KO: control 13.0 ± 5.4 SEM, Y27632 20.8 ± 12.1 SEM, EHT 1864 1.2 ± 0.7 SEM; *p ≤ 0.05) (Figures 6K,M). The results for PDL (Figures 6J,L) have already been described in the context of laminin-1 (Figures 3J,L). Taken together, our results indicate the importance of Rac1 for OPC migration on the TnC substrate, whereas RhoA signaling may rather inhibit the OPC migration in this context.

## Discussion

### Summary

We have shown that the ablation of the guanine nucleotide exchange factor Vav3 leads to a faster migration of OPCs on TnC *in vitro*. Other tested ECM molecules (Ln1, Ln2, TnR) did not indicate a Vav3 dependency. Further analysis of the number of migrated cells and the halos formed by migrating cells did not reveal significant alterations. However, we saw a clear involvement of the RhoA pathway after blockage of ROCK on PDL *in vitro*. Concerning TnC we observed an involvement of Rac1 in mediating migration behavior. These results suggest an important role of the GEF Vav3 in TnC-mediated migration. In addition, previous studies have shown that Vav3 modulates OPC differentiation and supports remyelination in white matter lesions (Ulc et al., 2019). Also, this GEF seems to limit dendritic development of hippocampal neurons, as demonstrated in an indirect co-culture system (Wegrzyn et al., 2021).

### Vav3 intervenes in the TnC-mediated migration of progenitor cells along Rac1 GTPases

It has already been published that Vav3 activates RhoA, RhoG, and Rac1 (Movilla and Bustelo, 1999; Aoki et al., 2005; Toumaniantz et al., 2009). RhoA and Rac1 are two of the most extensively studied Rho-family members. The function of RhoA was demonstrated by viral over-expression of dominant-negative (DN) and constitutively active (CA) Rho constructs in cultured primary oligodendrocytes. It has been shown that expression of CA-Rac1 induces oligodendrocyte process outgrowth, whereas CA-RhoA inhibits oligodendrocyte differentiation (Wolf et al., 2001; Liang et al., 2004). Additionally, it has been reported that RhoA is down-regulated during differentiation of primary oligodendrocytes (Liang et al., 2004; Mi et al., 2005) and that lysophosphatidic acid-mediated RhoA activation induces process retraction in OPCs (Dawson et al., 2003; Rajasekharan et al., 2010). On the other hand, the inactivation of RhoA enhances the differentiation and myelination in oligodendrocytes (Zhao et al., 2013). Moreover, FRET analysis revealed that there is a significant downregulation of Cdc42 and RhoA activity in Vav3-deficient oligodendrocytes, which is one of the reasons for a delayed myelination by these oligodendrocytes (Ulc et al., 2019). This indicates that Vav3 is a regulator for oligodendrocyte maturation and differentiation as well as for neuronal complexity, which could be shown using an indirect co-culture system of hippocampal neurons (Ulc et al., 2019; Wegrzyn et al., 2020). With regard to the differentiation of oligodendrocytes it has been reported that Rac1 is an important mediator of the oligodendrocyte actin cytoskeleton through the Wnt signaling pathway *via* Dishevelled associated activator of morphogenesis 2 (Daam2) (Cristobal et al., 2022).

OPCs are motile cells that migrate from their sites of origin towards territories that are bound for myelination (Kessaris et al., 2006; Bergles and Richardson, 2016; Tsai et al., 2016). The migration process requires the formation of distinct actin-based membrane protrusions such as lamellipodia and ruffles or filopodia and is controlled by several transforming proteins, like the actin filament-binding protein nexilin or the growth factor *β* (Small et al., 2002). Small GTPases of the Rho-family are key molecular switches that regulate the formation of cellular protrusions (Hall, 1998). Previous studies have shown an involvement of Vav3 in the migration of vascular smooth muscle cells, the granule cells of the cerebellum and in cancer cell migration (Toumaniantz et al., 2009; Quevedo et al., 2010; Ojala et al., 2020). In agreement with these reports Vav3-deficient OPCs displayed an elevated migration speed in the oligosphere migration assay on TnC (Figure 4). An increase in velocity of about 56.5% compared to wild-type OPCs could be measured. Previously it was found that TnC has anti-adhesive and anti-migratory effects on OPC motility (Bartsch et al., 1994; Kiernan et al., 1996; Wiemann et al., 2020; Bauch et al., 2022). Interestingly, both tenascins interfere with RhoA activation in OPCs, which should counteract the assumed anti-migratory effect of this GTPase (Czopka et al., 2009; Ulc et al., 2019). Similarly, ablation of Vav3 should reduce RhoA activation as well. Consequently, our data revealed an accelerated motility of *Vav3* ^−/−^ OPCs on TnC compared to *Vav3* ^+/+^ OPCs. These data concur to attribute a role of Vav3 to the intracellular mediation of TnC-signaling on OPC migration (Moritz et al., 2008).

In further ECM-based time-lapse migration assays we found substrate-dependent alterations in comparison to the control on Ln1, Ln2, and TnR. These findings were in line with previous studies that revealed a stimulated motility of OPCs on Ln1 and Ln2 (Frost et al., 1996; Milner et al., 1996; Kang and Yao, 2022). With regard to TnR previous studies focused on neural stem progenitor cell (NSPC) migration with the model of neurospheres, where two isoforms of TnR were indicated to inhibit cell motility (Huang et al., 2009). Also, the examination of the migration behavior of oligosphere-derived OPCs in our approach revealed at best a marginal mobility of OPCs on the TnR substrate compared to our observations with laminins. A slight increase compared to PDL can be explained by overall anti-adhesive properties compared to the control condition PDL. This effect might be explained by dose-dependent events and different influences of TnR on neural and glial cell types (Roll and Faissner, 2019; Bauch et al., 2022). However, while the substrates influenced the migration behavior, Vav3 did not seem to possess a role in mediating this cellular process on laminins and TnR.

It is of interest that Vav2 and Vav3 share a high sequence homology (Citterio et al., 2012) and synergize in regulating the number and functional status of hair follicle bulge stem cells (Lorenzo-Martin et al., 2022). This raises the possibility that Vav2 might compensate for Vav3 ablation in the experimental paradigm described in this study (Citterio et al., 2012; Bustelo, 2014; Wegrzyn et al., 2020). Along these lines, it has been postulated that Vav2 is a plausible candidate for the compensation of *Vav3* knockout in the retina (Katzav, 2009; Ulc et al., 2017). On the other hand, evidence has been reported that these molecules do not serve completely redundant functions (Citterio et al., 2012; Bustelo, 2014; Wegrzyn et al., 2020). Experiments analyzing the transcriptomic alterations consequent to the ablation of individual Vav proteins showed that the deletion resulted in distinct stable transcriptomic alterations in macrophages, indicating that each member of Vav in macrophages is functionally nonredundant (Huang et al., 2019). Also, Vav2 did not compensate the absence of Vav3 in a model of hippocampal neuron differentiation (Wegrzyn et al., 2020). In this context, it is worthwhile to consider the expression of Vav-message in the nervous system in more detail. Indeed, extensive transcriptome studies have been published based on carefully purified neural cell populations. According to these studies, Vav2 message is primarily expressed in microglia/macrophage populations of the CNS. In contrast and in accordance with our results Vav3 abounds in the astrocytes and OPCs (Zhang et al., 2014; Zhang et al., 2016). This is in agreement with our functional studies, as the increased migration on TnC of *Vav3* ^*−/−*^ OPCs could not be further augmented by the additional pharmacological blockade of the RhoA pathway, which would be expected if Vav2 were still to stimulate RhoA. This clearly speaks against a residual activity of Vav2 in the *Vav3*-deleted OPCs and hence against a compensation of Vav3-elimination by Vav2 in this situation.

Analyzing the effect of inhibiting RhoA and Rac1 pathway, the RhoA pathway occurred to have a suppressive impact on OPC migration as its blockage led to an increased motility or migration behavior of the cells. Previous studies have illustrated an opposing connection between RhoA and Rac1, whereupon RhoA is able to directly inhibit Rac1 and in contrast Rac1 can block RhoA *via* its effector WAVE2 (Parri and Chiarugi, 2010). However, Rac1 seems to be an important player concerning OPC migration, which could also be confirmed in a model of Ankylosing spondylitis (AS) (Xiao et al., 2013; Cui et al., 2022) and so we conclude that inhibition of RhoA released Rac1 from blockage by RhoA and therefore intensified the motility of OPCs. Furthermore, blockage of Rac1 caused significantly decreased migration parameters (speed, halos, number of migrating cells) and therefore supports the idea of a Rac1-mediated migration behavior of OPCs on the different ECM substrates.

Previous findings have shown that one pathway of Rac1 activation involves the Tiam1/Rac1/ERK signaling pathway (Xiao et al., 2013). Some effects of Rac1 activation are mediated by Pak1 and the effectors LIM and cofilin (Yang et al., 1998; Edwards et al., 1999; Toumaniantz et al., 2009). The most promising signaling partners for an ECM-based Rac1-activation are the integrin-receptors, especially in combination with discoidin domain receptors (DDRs) 1 and 2 it could be shown that they promote cell adhesion and migration on collagens (Borza et al., 2022). Exclusively about OPCs, it could be shown that especially the αvβ1 integrin-receptor is a promising candidate, because it is implicated in OPC migration processes (Milner et al., 1996). In this context, the small GTPase RhoG, which has not been studied extensively so far, might also be involved, because it is activated by the Vav3-GEF (Movilla and Bustelo, 1999). In support of this idea, it has been observed that RhoG is able to activate Rac1 *via* ELMO and Dock180 (Cote and Vuori, 2007). Whether RhoG is involved in the migration of OPCs remains to be clarified.

In conclusion, our observations provide new insights into the potential roles of the Rho-GEF Vav3 in the migration of OPCs. So far, the data suggest that this tyrosine-kinase-regulated GEF influences the migration of OPCs on TnC and this effect appears to be Rac1-dependent. This might reflect that Vav3 is part of signaling pathways that regulate the response to membrane-based tyrosine kinase receptors that mediate growth factor effects on progenitor cell populations.

## Data Availability

The raw data supporting the conclusion of this article will be made available by the authors, without undue reservation.

## References

[B1] Aoki K. Nakamura T. Fujikawa K. Matsuda M. (2005). Local phosphatidylinositol 3, 4, 5-trisphosphate accumulation recruits Vav2 and Vav3 to activate Rac1/Cdc42 and initiate neurite outgrowth in nerve growth factor-stimulated PC12 cells. Mol. Biol. Cell 16 (5), 2207–2217. 10.1091/mbc.E04-10-0904 15728722 PMC1087229

[B2] Barateiro A. Fernandes A. (2014). Temporal oligodendrocyte lineage progression: *In vitro* models of proliferation, differentiation and myelination. Biochim. Biophys. Acta 1843 (9), 1917–1929. 10.1016/j.bbamcr.2014.04.018 24768715

[B3] Bartsch U. Faissner A. Trotter J. Dörries U. Bartsch S. Mohajeri H. (1994). Tenascin demarcates the boundary between the myelinated and nonmyelinated part of retinal ganglion cell axons in the developing and adult mouse. J. Neurosci. 14 (8), 4756–4768. 10.1523/jneurosci.14-08-04756.1994 7519256 PMC6577191

[B5] Bauch J. Vom Ort S. Ulc A. Faissner A. (2022). Tenascins interfere with remyelination in an *ex vivo* cerebellar explant model of demyelination. Front. Cell Dev. Biol. 10, 819967. 10.3389/fcell.2022.819967 35372366 PMC8965512

[B6] Bergles D. E. Richardson W. D. (2016). Oligodendrocyte development and plasticity. Cold Spring Harb. Perspect. Biol. 8 (2), a020453. 10.1101/cshperspect.a020453 PMC4743079 26492571

[B7] Borza C. M. Bolas G. Zhang X. Browning Monroe M. B. Zhang M. Z. Meiler J. (2022). The collagen receptor discoidin domain receptor 1b enhances integrin β1-mediated cell migration by interacting with talin and promoting Rac1 activation. Front. Cell Dev. Biol. 10, 836797. 10.3389/fcell.2022.836797 35309920 PMC8928223

[B8] Bos J. L. Rehmann H. Wittinghofer A. (2007). GEFs and GAPs: Critical elements in the control of small G proteins. Cell 129 (5), 865–877. 10.1016/j.cell.2007.05.018 17540168

[B9] Bustelo X. R. Ledbetter J. A. Barbacid M. (1992). Product of vav proto-oncogene defines a new class of tyrosine protein kinase substrates. Nature 356 (6364), 68–71. 10.1038/356068a0 1311423

[B10] Bustelo X. R. (2014). Vav family exchange factors: An integrated regulatory and functional view. Small GTPases 5 (2), 9. 10.4161/21541248.2014.973757 25483299 PMC4601183

[B11] Bustelo X. R. (2001). Vav proteins, adaptors and cell signaling. Oncogene 20 (44), 6372–6381. 10.1038/sj.onc.1204780 11607839

[B12] Chiariello M. Marinissen M. J. Gutkind J. S. (2001). Regulation of c-myc expression by PDGF through Rho GTPases. Nat. Cell Biol. 3 (6), 580–586. 10.1038/35078555 11389443

[B13] Citterio C. Menacho-Márquez M. García-Escudero R. Larive R. M. Barreiro O. Sánchez-Madrid F. (2012). The rho exchange factors vav2 and vav3 control a lung metastasis-specific transcriptional program in breast cancer cells. Sci. Signal. 5 (244), ra71. 10.1126/scisignal.2002962 23033540

[B14] Côté J. F. Vuori K. (2007). GEF what? Dock180 and related proteins help rac to polarize cells in new ways. Trends Cell Biol. 17 (8), 383–393. 10.1016/j.tcb.2007.05.001 17765544 PMC2887429

[B15] Cristobal C. D. Wang C. Y. Zuo Z. Smith J. A. Lindeke-Myers A. Bellen H. J. (2022). Daam2 regulates myelin structure and the oligodendrocyte actin cytoskeleton through Rac1 and gelsolin. J. Neurosci. 42 (9), 1679–1691. 10.1523/JNEUROSCI.1517-21.2022 35101966 PMC8896627

[B16] Cui H. Li Z. Chen S. Li X. Chen D. Wang J. (2022). CXCL12/CXCR4-Rac1-mediated migration of osteogenic precursor cells contributes to pathological new bone formation in ankylosing spondylitis. Sci. Adv. 8 (14), eabl8054. 10.1126/sciadv.abl8054 35385310 PMC8986111

[B18] Czopka T. Von Holst A. Schmidt G. Ffrench-Constant C. Faissner A. (2009). Tenascin C and tenascin R similarly prevent the formation of myelin membranes in a RhoA-dependent manner, but antagonistically regulate the expression of myelin basic protein via a separate pathway. Glia 57 (16), 1790–1801. 10.1002/glia.20891 19459213

[B19] Dawson J. Hotchin N. Lax S. Rumsby M. (2003). Lysophosphatidic acid induces process retraction in CG-4 line oligodendrocytes and oligodendrocyte precursor cells but not in differentiated oligodendrocytes. J. Neurochem. 87 (4), 947–957. 10.1046/j.1471-4159.2003.02056.x 14622125

[B20] Edwards D. C. Sanders L. C. Bokoch G. M. Gill G. N. (1999). Activation of LIM-kinase by Pak1 couples Rac/Cdc42 GTPase signalling to actin cytoskeletal dynamics. Nat. Cell Biol. 1 (5), 253–259. 10.1038/12963 10559936

[B76] Faissner A. Kruse J. (1990). J1/tenascin is a repulsive substrate for central nervous system neurons. Neuron. 5, 627–637. 10.1016/0896-6273(90)90217-4 1699568

[B21] Ferent J. Zimmer C. Durbec P. Ruat M. Traiffort E. (2013). Sonic Hedgehog signaling is a positive oligodendrocyte regulator during demyelination. J. Neurosci. 33 (5), 1759–1772. 10.1523/JNEUROSCI.3334-12.2013 23365216 PMC6619133

[B22] Frost E. Kiernan B. W. Faissner A. ffrench-Constant C. (1996). Regulation of oligodendrocyte precursor migration by extracellular matrix: Evidence for substrate-specific inhibition of migration by tenascin-C. Dev. Neurosci. 18 (4), 266–273. 10.1159/000111416 8911766

[B23] Govek E. E. Newey S. E. Van Aelst L. (2005). The role of the Rho GTPases in neuronal development. Genes Dev. 19 (1), 1–49. 10.1101/gad.1256405 15630019

[B24] Hall A. (1998). Rho GTPases and the actin cytoskeleton. Science 279 (5350), 509–514. 10.1126/science.279.5350.509 9438836

[B25] Hornstein I. Alcover A. Katzav S. (2004). Vav proteins, masters of the world of cytoskeleton organization. Cell. Signal. 16 (1), 1–11. 10.1016/s0898-6568(03)00110-4 14607270

[B26] Huang R. Guo G. Lu L. Fu R. Luo J. Liu Z. (2019). The three members of the Vav family proteins form complexes that concur to foam cell formation and atherosclerosis. J. Lipid Res. 60 (12), 2006–2019. 10.1194/jlr.M094771 31570505 PMC6889716

[B27] Huang W. Zhang L. Niu R. Liao H. (2009). Tenascin-R distinct domains modulate migration of neural stem/progenitor cells *in vitro* . Vitro Cell. Dev. Biol. Anim. 45 (1-2), 10–14. 10.1007/s11626-008-9145-6 18855077

[B28] Jaffe A. B. Hall A. (2005). Rho GTPases: Biochemistry and biology. Annu. Rev. Cell Dev. Biol. 21, 247–269. 10.1146/annurev.cellbio.21.020604.150721 16212495

[B29] Kang M. Yao Y. (2022). Laminin regulates oligodendrocyte development and myelination. Glia 70 (3), 414–429. 10.1002/glia.24117 34773273 PMC8817735

[B30] Katzav S. (2009). Vav1: A hematopoietic signal transduction molecule involved in human malignancies. Int. J. Biochem. Cell Biol. 41 (6), 1245–1248. 10.1016/j.biocel.2008.11.006 19100858

[B31] Kessaris N. Fogarty M. Iannarelli P. Grist M. Wegner M. Richardson W. D. (2006). Competing waves of oligodendrocytes in the forebrain and postnatal elimination of an embryonic lineage. Nat. Neurosci. 9 (2), 173–179. 10.1038/nn1620 16388308 PMC6328015

[B32] Kiernan B. W. Götz B. Faissner A. ffrench-Constant C. (1996). Tenascin-C inhibits oligodendrocyte precursor cell migration by both adhesion-dependent and adhesion-independent mechanisms. Mol. Cell. Neurosci. 7 (4), 322–335. 10.1006/mcne.1996.0024 8793866

[B33] Kriegstein A. Alvarez-Buylla A. (2009). The glial nature of embryonic and adult neural stem cells. Annu. Rev. Neurosci. 32, 149–184. 10.1146/annurev.neuro.051508.135600 19555289 PMC3086722

[B34] Liang X. Draghi N. A. Resh M. D. (2004). Signaling from integrins to Fyn to Rho family GTPases regulates morphologic differentiation of oligodendrocytes. J. Neurosci. 24 (32), 7140–7149. 10.1523/JNEUROSCI.5319-03.2004 15306647 PMC6729178

[B35] Lorenzo-Martín L. F. Menacho-Márquez M. Fernández-Parejo N. Rodríguez-Fdez S. Pascual G. Abad A. (2022). The Rho guanosine nucleotide exchange factors Vav2 and Vav3 modulate epidermal stem cell function. Oncogene 41 (24), 3341–3354. 10.1038/s41388-022-02341-7 35534539 PMC9187518

[B36] Luft V. Reinhard J. Shibuya M. Fischer K. D. Faissner A. (2015). The guanine nucleotide exchange factor Vav3 regulates differentiation of progenitor cells in the developing mouse retina. Cell Tissue Res. 359 (2), 423–440. 10.1007/s00441-014-2050-2 25501893

[B37] Marcoux N. Vuori K. (2003). EGF receptor mediates adhesion-dependent activation of the rac GTPase: A role for phosphatidylinositol 3-kinase and Vav2. Oncogene 22 (38), 6100–6106. 10.1038/sj.onc.1206712 12955089

[B38] Margolis B. Hu P. Katzav S. Li W. Oliver J. M. Ullrich A. (1992). Tyrosine phosphorylation of vav proto-oncogene product containing SH2 domain and transcription factor motifs. Nature 356 (6364), 71–74. 10.1038/356071a0 1531699

[B39] Melamed I. Patel H. Brodie C. Gelfand E. W. (1999). Activation of Vav and Ras through the nerve growth factor and B cell receptors by different kinases. Cell. Immunol. 191 (2), 83–89. 10.1006/cimm.1998.1402 9973529

[B40] Mi S. Miller R. H. Lee X. Scott M. L. Shulag-Morskaya S. Shao Z. (2005). LINGO-1 negatively regulates myelination by oligodendrocytes. Nat. Neurosci. 8 (6), 745–751. 10.1038/nn1460 15895088

[B41] Milner R. Edwards G. Streuli C. Ffrench-Constant C. (1996). A role in migration for the alpha V beta 1 integrin expressed on oligodendrocyte precursors. J. Neurosci. 16 (22), 7240–7252. 10.1523/jneurosci.16-22-07240.1996 8929432 PMC6578950

[B42] Moores S. L. Selfors L. M. Fredericks J. Breit T. Fujikawa K. Alt F. W. (2000). Vav family proteins couple to diverse cell surface receptors. Mol. Cell. Biol. 20 (17), 6364–6373. 10.1128/mcb.20.17.6364-6373.2000 10938113 PMC86111

[B43] Moritz S. Lehmann S. Faissner A. von Holst A. (2008). An induction gene trap screen in neural stem cells reveals an instructive function of the niche and identifies the splicing regulator sam68 as a tenascin-C-regulated target gene. Stem Cells 26 (9), 2321–2331. 10.1634/stemcells.2007-1095 18617690

[B44] Movilla N. Bustelo X. R. (1999). Biological and regulatory properties of Vav-3, a new member of the Vav family of oncoproteins. Mol. Cell. Biol. 19 (11), 7870–7885. 10.1128/mcb.19.11.7870 10523675 PMC84867

[B45] Nave K. A. Werner H. B. (2014). Myelination of the nervous system: Mechanisms and functions. Annu. Rev. Cell Dev. Biol. 30, 503–533. 10.1146/annurev-cellbio-100913-013101 25288117

[B46] Nobes C. D. Hall A. (1995). Rho, rac and cdc42 GTPases: Regulators of actin structures, cell adhesion and motility. Biochem. Soc. Trans. 23 (3), 456–459. 10.1042/bst0230456 8566347

[B47] Ojala V. K. Knittle A. M. Kirjalainen P. Merilahti J. A. M. Kortesoja M. Tvorogov D. (2020). The guanine nucleotide exchange factor VAV3 participates in ERBB4-mediated cancer cell migration. J. Biol. Chem. 295 (33), 11559–11571. 10.1074/jbc.RA119.010925 32561640 PMC7450113

[B48] Parri M. Chiarugi P. (2010). Rac and Rho GTPases in cancer cell motility control. Cell Commun. Signal. 8, 23. 10.1186/1478-811X-8-23 20822528 PMC2941746

[B49] Pedraza C. E. Monk R. Lei J. Hao Q. Macklin W. B. (2008). Production, characterization, and efficient transfection of highly pure oligodendrocyte precursor cultures from mouse embryonic neural progenitors. Glia 56 (12), 1339–1352. 10.1002/glia.20702 18512250 PMC4395472

[B77] Pesheva P. Spiess E. Schachner M. (1989). J1-160 and J1-180 are oligodendrocyte-secreted nonpermissive substrates for cell adhesion. J. Cell. Biol. 109 (4 Pt 1), 1765–1778. 10.1083/jcb.109.4.1765 2477380 PMC2115782

[B50] Petersen M. A. Ryu J. K. Chang K. J. Etxeberria A. Bardehle S. Mendiola A. S. (2017). Fibrinogen activates BMP signaling in oligodendrocyte progenitor cells and inhibits remyelination after vascular damage. Neuron 96 (5), 1003–1012. 10.1016/j.neuron.2017.10.008 29103804 PMC5851281

[B51] Quevedo C. Sauzeau V. Menacho-Márquez M. Castro-Castro A. Bustelo X. R. (2010). Vav3-deficient mice exhibit a transient delay in cerebellar development. Mol. Biol. Cell 21 (6), 1125–1139. 10.1091/mbc.e09-04-0292 20089829 PMC2836963

[B52] Rajasekharan S. Bin J. M. Antel J. P. Kennedy T. E. (2010). A central role for RhoA during oligodendroglial maturation in the switch from netrin-1-mediated chemorepulsion to process elaboration. J. Neurochem. 113 (6), 1589–1597. 10.1111/j.1471-4159.2010.06717.x 20367748

[B53] Roll L. Faissner A. (2019). Tenascins in CNS lesions. Semin. Cell Dev. Biol. 89, 118–124. 10.1016/j.semcdb.2018.09.012 30287388

[B54] Sachdev P. Zeng L. Wang L. H. (2002). Distinct role of phosphatidylinositol 3-kinase and Rho family GTPases in Vav3-induced cell transformation, cell motility, and morphological changes. J. Biol. Chem. 277 (20), 17638–17648. 10.1074/jbc.M111575200 11884391

[B78] Schindelin J. Arganda-Carreras I. Frise E. Kaynig V. Longair M. Pietzsch T. (2012). Fiji: An open-source platform for biological-image analysis. Nat. Methods 9, 676–682. 10.1038/nmeth.2019 22743772 PMC3855844

[B79] Schneider C. A. Rasband W. S. Eliceiri K. W. (2012). NIH Image to ImageJ: 25 years of image analysis. Nat. Methods 9, 671–675. 10.1038/nmeth.2089 22930834 PMC5554542

[B55] Shutes A. Onesto C. Picard V. Leblond B. Schweighoffer F. Der C. J. (2007). Specificity and mechanism of action of EHT 1864, a novel small molecule inhibitor of Rac family small GTPases. J. Biol. Chem. 282 (49), 35666–35678. 10.1074/jbc.M703571200 17932039

[B56] Small J. V. Stradal T. Vignal E. Rottner K. (2002). The lamellipodium: Where motility begins. Trends Cell Biol. 12 (3), 112–120. 10.1016/s0962-8924(01)02237-1 11859023

[B57] Suzuki N. Hyodo M. Hayashi C. Mabuchi Y. Sekimoto K. Onchi C. (2019). Laminin α2, α4, and α5 chains positively regulate migration and survival of oligodendrocyte precursor cells. Sci. Rep. 9 (1), 19882. 10.1038/s41598-019-56488-7 31882770 PMC6934537

[B58] Toumaniantz G. Ferland-McCollough D. Cario-Toumaniantz C. Pacaud P. Loirand G. (2009). The Rho protein exchange factor Vav3 regulates vascular smooth muscle cell proliferation and migration. Cardiovasc. Res. 86 (1), 131–140. 10.1093/cvr/cvp387 19969623

[B59] Tsai H. H. Niu J. Munji R. Davalos D. Chang J. Zhang H. (2016). Oligodendrocyte precursors migrate along vasculature in the developing nervous system. Science 351 (6271), 379–384. 10.1126/science.aad3839 26798014 PMC5472053

[B60] Turner M. Billadeau D. D. (2002). VAV proteins as signal integrators for multi-subunit immune-recognition receptors. Nat. Rev. Immunol. 2 (7), 476–486. 10.1038/nri840 12094222

[B61] Uddin S. Katzav S. White M. F. Platanias L. C. (1995). Insulin-dependent tyrosine phosphorylation of the vav protooncogene product in cells of hematopoietic origin. J. Biol. Chem. 270 (13), 7712–7716. 10.1074/jbc.270.13.7712 7535775

[B62] Ulc A. Gottschling C. Schäfer I. Wegrzyn D. van Leeuwen S. Luft V. (2017). Involvement of the guanine nucleotide exchange factor Vav3 in central nervous system development and plasticity. Biol. Chem. 398 (5-6), 663–675. 10.1515/hsz-2016-0275 28214347

[B63] Ulc A. Zeug A. Bauch J. van Leeuwen S. Kuhlmann T. Ffrench-Constant C. (2019). The guanine nucleotide exchange factor Vav3 modulates oligodendrocyte precursor differentiation and supports remyelination in white matter lesions. Glia 67 (2), 376–392. 10.1002/glia.23548 30450647

[B64] Valério-Gomes B. Guimarães D. M. Szczupak D. Lent R. (2018). The absolute number of oligodendrocytes in the adult mouse brain. Front. Neuroanat. 12, 90. 10.3389/fnana.2018.00090 30425626 PMC6218541

[B65] Wegrzyn D. Wegrzyn C. Tedford K. Fischer K. D. Faissner A. (2020). Deletion of the nucleotide exchange factor Vav3 enhances axonal complexity and synapse formation but tampers activity of hippocampal neuronal networks *in vitro* . Int. J. Mol. Sci. 21 (3), E856. 10.3390/ijms21030856 PMC7037001 32013053

[B66] Wegrzyn D. Zokol J. Faissner A. (2021). Vav3-Deficient astrocytes enhance the dendritic development of hippocampal neurons in an indirect Co-culture system. Front. Cell. Neurosci. 15, 817277. 10.3389/fncel.2021.817277 35237130 PMC8882586

[B67] Wiemann S. Reinhard J. Reinehr S. Cibir Z. Joachim S. C. Faissner A. (2020). Loss of the extracellular matrix molecule tenascin-C leads to absence of reactive gliosis and promotes anti-inflammatory cytokine expression in an autoimmune glaucoma mouse model. Front. Immunol. 11, 566279. 10.3389/fimmu.2020.566279 33162981 PMC7581917

[B68] Wolf R. M. Wilkes J. J. Chao M. V. Resh M. D. (2001). Tyrosine phosphorylation of p190 RhoGAP by Fyn regulates oligodendrocyte differentiation. J. Neurobiol. 49 (1), 62–78. 10.1002/neu.1066 11536198

[B69] Xiao L. Hu C. Yang W. Guo D. Li C. Shen W. (2013). NMDA receptor couples Rac1-GEF Tiam1 to direct oligodendrocyte precursor cell migration. Glia 61 (12), 2078–2099. 10.1002/glia.22578 24123220

[B70] Yang N. Higuchi O. Ohashi K. Nagata K. Wada A. Kangawa K. (1998). Cofilin phosphorylation by LIM-kinase 1 and its role in Rac-mediated actin reorganization. Nature 393 (6687), 809–812. 10.1038/31735 9655398

[B71] Yu B. Martins I. R. Li P. Amarasinghe G. K. Umetani J. Fernandez-Zapico M. E. (2010). Structural and energetic mechanisms of cooperative autoinhibition and activation of Vav1. Cell 140 (2), 246–256. 10.1016/j.cell.2009.12.033 20141838 PMC2825156

[B72] Zhang Y. Chen K. Sloan S. A. Bennett M. L. Scholze A. R. O'Keeffe S. (2014). An RNA-sequencing transcriptome and splicing database of glia, neurons, and vascular cells of the cerebral cortex. J. Neurosci. 34 (36), 11929–11947. 10.1523/JNEUROSCI.1860-14.2014 25186741 PMC4152602

[B73] Zhang Y. Sloan S. A. Clarke L. E. Caneda C. Plaza C. A. Blumenthal P. D. (2016). Purification and characterization of progenitor and mature human astrocytes reveals transcriptional and functional differences with mouse. Neuron 89 (1), 37–53. 10.1016/j.neuron.2015.11.013 26687838 PMC4707064

[B74] Zhao C. F. Liu Y. Que H. P. Yang S. G. Liu T. Liu Z. Q. (2013). Rnh1 promotes differentiation and myelination via RhoA in oligodendrocytes. Cell Tissue Res. 353 (3), 381–389. 10.1007/s00441-013-1625-7 23624614

[B75] Zheng Y. (2001). Dbl family guanine nucleotide exchange factors. Trends biochem. Sci. 26 (12), 724–732. 10.1016/s0968-0004(01)01973-9 11738596

